# Low rates of nucleoside reverse transcriptase inhibitor and nonnucleoside reverse transcriptase inhibitor drug resistance in Botswana

**DOI:** 10.1097/QAD.0000000000002166

**Published:** 2019-04-04

**Authors:** Sikhulile Moyo, Simani Gaseitsiwe, Melissa Zahralban-Steele, Dorcas Maruapula, Tapiwa Nkhisang, Baitshepi Mokaleng, Terence Mohammed, Tsotlhe R. Ditlhako, Ontlametse T. Bareng, Thatayaone P. Mokgethi, Erik van Widenfelt, Molly Pretorius-Holme, Madisa O. Mine, Elliot Raizes, Etienne Kadima Yankinda, Kathleen E. Wirth, Tendani Gaolathe, Joseph M. Makhema, Shahin Lockman, Max Essex, Vlad Novitsky

**Affiliations:** a Botswana Harvard AIDS Institute Partnership, Gaborone, Botswana; b Department of Immunology and Infectious Diseases, Harvard T.H. Chan School of Public Health, Boston, USA; c Ministry of Health and Wellness, Gaborone, Botswana; d U.S. Centers for Disease Control and Prevention, Atlanta; e Harvard Medical School; f Division of Infectious Diseases, Department of Medicine, Brigham and Women's Hospital, Boston, USA.

**Keywords:** Botswana, HIV-1 drug resistance, nonnucleoside reverse transcriptase inhibitors, nucleoside reverse transcriptase inhibitors

## Abstract

**Background:**

Scale-up of antiretroviral therapy (ART) and introduction of treat-all strategy necessitates population-level monitoring of acquired HIV drug resistance (ADR) and pretreatment drug resistance (PDR) mutations.

**Methods:**

Blood samples were collected from 4973 HIV-positive individuals residing in 30 communities across Botswana who participated in the Botswana Combination Prevention Project (BCPP) in 2013–2018. HIV sequences were obtained by long-range HIV genotyping. Major drug-resistance mutations (DRMs) and surveillance drug resistance mutations (SDRMs) associated with nucleoside reverse transcriptase inhibitors (NRTI) and nonnucleoside reverse transcriptase inhibitors (NNRTI) were analyzed according to the Stanford University HIV Drug Resistance Database. Viral sequences were screened for G-to-A hypermutations. A threshold of 2% was used for hypermutation adjustment. Viral suppression was considered at HIV-1 RNA load ≤400 copies/ml.

**Results:**

Among 4973 participants with HIV-1C sequences, ART data were available for 4927 (99%) including 3858 (78%) on ART. Among those on ART, 3435 had viral load data and 3297 (96%) were virologically suppressed. Among 1069 (22%) HIV-infected individuals not on ART, we found NRTI-associated and NNRTI-associated SDRMs were found in 1.5% (95% confidence interval [CI] 1.0–2.5%) and 2.9% (95% CI 2.0–4.2%), respectively. Of the 138 (4%) of individuals who had detectable HIV-1 RNA, we found NRTI-associated and NNRTI-associated drug resistance mutations in 16% (95% CI 10–25%) and 33% (95% CI 25–42%), respectively.

**Conclusion:**

We found a low prevalence of NRTI-associated and NNRTI-associated PDR-resistance mutations among residents of rural and peri-urban communities across Botswana. However, individuals on ART with detectable virus had ADR NRTI and NNRTI mutations above 15%.

## Abstract 2

Supplemental Digital Content is available in the text

## Introduction

Antiretroviral therapy (ART) coverage has increased globally with over 21 million people now receiving lifesaving treatment [1]. Sub-Saharan Africa has achieved the greatest increase in ART coverage [2]. Many countries have adopted the WHO recommendation to initiate ART in all HIV-infected individuals [3] regardless of CD4^+^ T-cell count.

Although ART has drastically reduced HIV-related mortality and morbidity, global sustainable scale-up of ART could lead to emergence and spread of drug-resistant HIV strains [3,4]. A successful scale-up of ART has been associated with an increase in pretreatment drug resistance (PDR) in low-resource settings [4]. A recent WHO report showed that the prevalence of PDR to nonnucleoside reverse transcriptase inhibitors (NNRTIs) surpassed 10% in 6 of 11 countries surveyed [5]. Reaching the WHO 10% threshold of drug resistance might require changing of the first-line ART regimen countrywide. A recent meta-analysis across 63 countries [4] revealed overall increase (up to 23% in Southern Africa) of PDR to NNRTIs. Drug-resistant HIV strains limit treatment options, presenting a threat to effective scale-up of ART and getting HIV infection under control by 2030 [6]. Thus, population-level monitoring of HIV drug resistance (HIVDR) is critical.

The estimated number of people living with HIV in Botswana by end of 2017 was 380 000 [7]. Botswana introduced a national ART programme in January 2002 and implemented test-and-treat (TREAT ALL) policy in June 2016 [8]. Current ART coverage in Botswana is estimated at 84% [9]. Low levels of HIVDR were detected among a few sub-populations in the capital city and surrounding villages in 2001 [10] and 2007 [11]. However, a recent study among treatment-naive pregnant women in Gaborone showed a sharp increase in the prevalence of overall transmitted drug resistance from 2.9% in 2012 to 9.7% 2015 [12], highlighting a need for on-going population-level monitoring of drug resistance. This study aimed to survey the prevalence of HIV-1 mutations associated with resistance to nucleoside reverse transcriptase inhibitors (NRTI) and NNRTIs among rural and peri-urban communities across Botswana.

## Methods

### Study population

Blood samples were collected from a random sample of HIV-positive participants aged 16–64 years in the Botswana Combination Prevention Project (BCPP) [13] who reside in 30 rural and peri-urban communities across the North, Central, and Southern regions of Botswana. The HIV-positive status of participants was based on either written documentation provided (e.g. HIV test results, ART prescription) or HIV testing that was performed in the households according to the Botswana national guidelines by using two rapid HIV tests in parallel. Both HIV-positive participants receiving ART and not receiving ART were included in this study. For all participants who self-reported to be on ART, ART status was verified through documentation (e.g. prescriptions, clinical notes showing ART receipt, or possession of pills) [13]. Most of the participants on ART were receiving either tenofovir/emtricitabine/efavirenz (Atripla) or tenofovir/emtricitabine/nevirapine (Truvada/NVP) or zidovudine/lamivudine/efavirenz (Combivir/EFV) or zidovudine/lamivudine/nevirapine (Combivir/NVP), the first-line regimens in the national ART program in Botswana at the time BCPP study was conducted.

The study was conducted in accordance with the Declaration of Helsinki. The study received independent ethics committee/institutional review board approval from the Botswana Health Research Development Committee (HPDME 13/18/1) and the US Centers for Disease Control and Prevention (Protocol 6475). All participants provided written informed consent. Participants aged 16–18 years provided written assent (with parents or guardians providing written permission). The study is registered at ClinicalTrials.gov (NCT01965470).

### Near full-length HIV genotyping

Viral sequences were generated by a long-range HIV genotyping protocol described elsewhere [14] with minor modifications that included a reduced annealing temperature (58 °C instead of 62 °C) as a backup amplification strategy and using the first-round amplicon as a template for next-generation sequencing (NGS). Both viral RNA and proviral DNA templates were used for amplification, as the majority of participants were receiving ART. The NGS was performed by the BioPolimers Facility at Harvard Medical School (https://genome.med.harvard.edu/) and through collaboration with PANGEA HIV consortium [15] at the Welcome Trust Sanger Institute (Cambridge, UK; http://www.sanger.ac.uk/) with high-sequencing coverage and using Illumina platform MiSeq and HiSeq. A single consensus sequence represented population of viral quasispecies per each participant. Out of 4973 HIV sequences, 4964 (99.8%) were generated by NGS, whereas the first nine (0.2%) sequences in the dataset were generated by Sanger sequencing. Minor viral variants were not analyzed in this study.

### HIV-1 subtyping

Generated near full-length HIV sequences were subtyped by on-line tools REGA HIV-1 subtyping tool, ver. 3 [16] and COMET [17]. HIV-1 subtype C (HIV-1C) sequences accounted for more than 99% of screened specimens (data not shown). Thus, only HIV-1C sequences were included in this study.

### Analysis of drug resistance

NRTI-associated and NNRTI-associated resistance mutations were analyzed according to the lists of SDRMs and major DRMs at Stanford University HIV Drug Resistance Database [18,19]. The proportions of HIV-associated DRMs among viruses circulating in Botswana were estimated at a population level. 95% confidence intervals (CIs) were estimated accounting for clustering by communities.

### Apolipoprotein B mRNA editing enzyme, catalytic polypeptide-like-induced hypermutations

Viral sequences were screened for guanine-to-adenine transitions (G-to-A) apolipoprotein B mRNA editing enzyme, catalytic polypeptide-like-induced hypermutations using Hypermut [20] at the Los Alamos National Laboratory HIV Database (http://www.hiv.lanl.gov/). Adjustment for hypermutations was performed, as previously described [21]. The HIV-1C consensus sequence was used as a reference. The adjusted hypermutations accounted for actual sequence length of the analyzed HIV-1 *pol* gene. Viral sequences with adjusted hypermutation rate above 2% were considered to be hypermutated. We have generated a list of selected amino acid mutations associated with HIVDR and estimated their relevance to hypermutation (see Supplementary Table S1). For example, A-to-T, or D-to-N or E-to-K were considered to be relevant to hypermutation, whereas A-to-I, or D-to-E or E-to-A were considered as not relevant to hypermutation. DRMs in hypermutated sequences relevant to hypermutation were not counted toward drug-resistance, whereas mutations that are not relevant to hypermutation were counted.

### HIV-1 RNA quantification

The HIV-1 RNA load in plasma was quantified at the same time specimens were sequenced. Abbott m2000sp/Abbott m2000rt (Wiesbaden, Germany) was used for quantification of viral load. HIV-1 RNA more than 400 copies/ml was considered as detectable viral load.

## Results

The median age at enrollment of the participants included in this analysis study was 40 years [interquartile range (IQR) 33–47] and 72% were women. Among 4973 participants with HIV-1C sequences, 4927 (99%) had definitive ART status (either ‘On ART’ or ‘ART-naive’). The majority of these participants 3858 (78%) were on ART, whereas 1069 (22%) were ART-naive at the time of sampling. Among 3435 participants on ART with available HIV-1 RNA load data, 3297 (96%) were virologically suppressed at 400 copies/ml threshold and 3325 (97%) were virologically suppressed at 1000 copies/ml threshold. Among individuals not suppressed, the median viral load was 23 942 (Q1, Q3: 7450–75 337) copies/ml among ART-naive participants (*n* = 857) and 7351 (Q1, Q3: 1258–40 552) among those on ART (*n* = 138).

Among HIV-infected individuals not on ART (*n* = 1069), NRTI-associated and NNRTI- associated SDRMs were found in 1.5% (95% CI 1.0–2.53%) and 2.9% (95% CI 2.0–4.2%), respectively (Table 1). Among individuals on ART who were not suppressed (*n* = 138), NRTI-associated and NNRTI-associated DRMs were found in 15.9% (95% CI 9.8–24.8%) and 32.6% (95% CI 24.6–41.8%), respectively (Table 2).

**Table 1:** Participant characteristics and prevalence of pretreatment nucleoside reverse transcriptase inhibitor-drug and nonnucleoside reverse transcriptase inhibitor-drug-resistant mutations among antiretroviral-naive Botswana combination prevention program participants.

	PDR (SDRM) prevalence		
Group	*N*	NRTI, % (95% CI)	NNRTI, % (95% CI)
All participants	1069	1.5 (1.0–2.3)	2.9 (2.0–4.2)
Sex			
Males	734	1.5 (0.9–2.4)	3.0 (2.0–4.5)
Females	335	1.5 (0.4–4.4)	2.7 (1.5–4.7)
Age (years)			
<30	340	1.2 (0.4–3.0)	3.8 (2.2–6.7)
30–39	373	2.7 (1.6–4.6)	4.3 (2.4–7.5)
40–49	225	0.4 (0.1–3.3)	0.4 (0.1–2.8)
50+	131	0.8 (0.1–5.8)	0.8 (0.1–6.1)
Geographical region			
South	519	1.6 (0.7–3.5)	3.4 (2.2–5.5)
Central	140	1.2 (0.6–2.4)	1.5 (0.6–3.4)
North	410	2.3 (1.2–4.4)	3.8 (2.0–7.0)
Year of sampling			
2013–2015	763	1.7 (1.0–2.8)	3.0 (1.9–4.7)
2016–2018	306	1.0 (0.4–2.7)	2.6 (1.3–5.0)

CI, confidence intervals; NNRTI, nonnucleoside reverse transcriptase inhibitors; NRTI, nucleoside reverse transcriptase inhibitors; PDR, pretreatment drug resistance; SDRM, surveillance drug-resistant mutations.

**Table 2:** Participants characteristics and prevalence of acquired nucleoside reverse transcriptase inhibitor-drug resistant mutations and nonnucleoside reverse transcriptase inhibitor-drug resistant mutations among virologically unsuppressed participants on antiretroviral therapy.

	ADR (DRM) prevalence		
Group	*N*	NRTI, % (95% CI)	NNRTI, % (95% CI)
All participants	138	15.9 (9.8–24.8)	32.6 (24.6–41.8)
Sex			
Males	54	19.7 (10.1–33.0)	25.9 (16.4–38.4)
Females	84	11.1 (6.0–19.7)	36.9 (25.8–49.5)
Age (years)			
<30	42	25.8 (10.3–51.2)	48.4 (30.4–66.8)
30–39	49	13.7 (4.9–32.9)	27.5 (17.5–40.2)
40–49	29	6.9 (1.6–25.7)	17.2 (7.0–36.5)
50+	18	16.7 (5.0–43.4)	44.4 (26.1–64.4)
Geographical region			
South	39	17.9 (7.4–37.4)	23.1 (10.4–43.6)
Central	54	16.7 (8.2–30.7)	33.3 (24.0–44.2)
North	45	13.3 (4.7–32.3)	40.0 (7.9–56.7)
Year of sampling			
2013–2015	86	19.8 (11.8–31.2)	26.7 (18.9–36.4)
2016–2018	52	9.6 (3.5–23.7)	15.4 (6.7–31.5)

ADR, acquired drug resistance; CI, confidence intervals; DRM, drug-resistant mutations; NNRTI, nonnucleoside reverse transcriptase inhibitors; NRTI, nucleoside reverse transcriptase inhibitors.

**Fig. 1:**
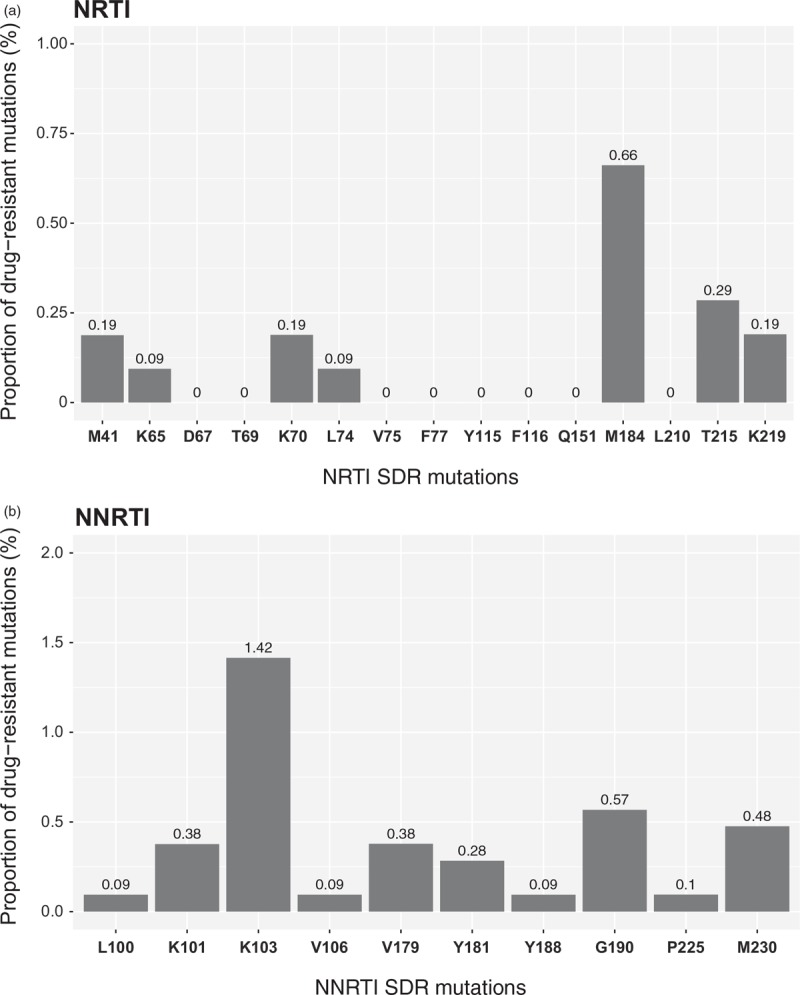
Proportion of surveillance drug-resistant mutations.

The overall distribution of NRTI-associated PDR among ART-naive BCPP participants is outlined in Fig. 1a. The most prevalent DRMs for NRTIs were M184 (0.66%). Prevalence of other NRTI-associated mutations was under 0.3%. Prevalence of NNRTI-associated PDR mutations among ART-naive BCPP participants is presented in Fig. 1b. Two NNRTI PDR mutations, K103 and G190, were observed at frequencies 1.42 and 0.57%, respectively, whereas other NNRTI-associated PDR mutations were at the level of less than 0.5%.

(a) Nucleoside reverse transcriptase inhibitors. The following amino acids substitutions were analyzed, as NRTI SDRMs: M41L; K65R/E/N; D67N/G/E; T69D; K70R/E/G/Q/N/T; L74V/I; V75I; F77L; Y115F; F116Y; Q151M; M184I/V; L210W; T215Y/F/S/C/D/E/I/V/A/L/N; K219Q/E. (b) Nucleoside reverse transcriptase inhibitors. The following amino acids substitutions were analyzed, as NNRTI SDRMs: L100I; K101P/E/H; K103N/S; V106M/A/I; V179D/F/T/L; Y181C/I/V; Y188L/H/C; G190A/S/E/Q; P225H; M230L/I. NRTI, nucleoside reverse transcriptase inhibitors; SDRM, surveillance drug resistant mutations.

**Fig. 2:**
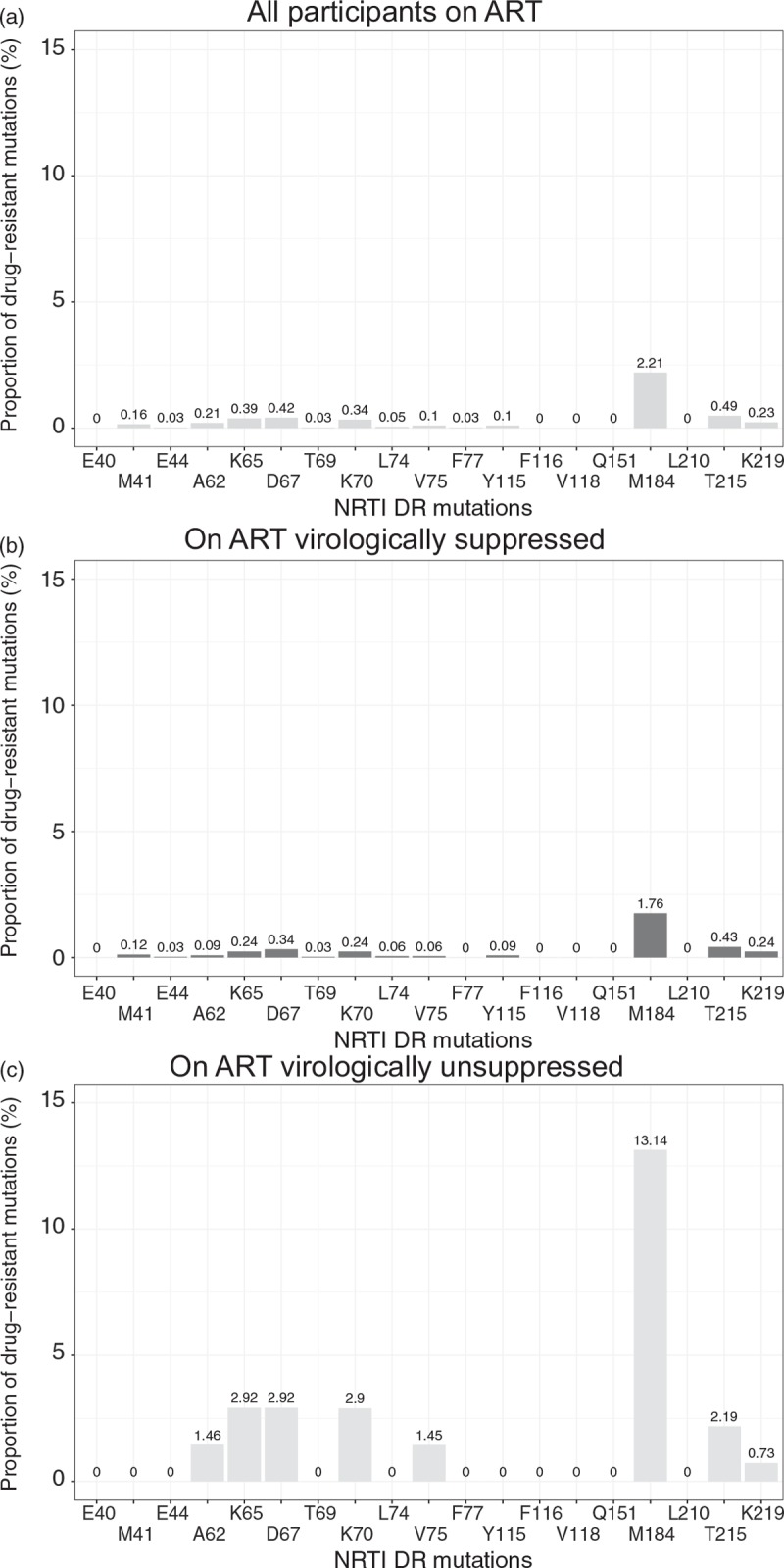
Proportion of nucleoside reverse transcriptase inhibitor drug-resistant mutations among study participants on antiretroviral therapy.

**Fig. 3:**
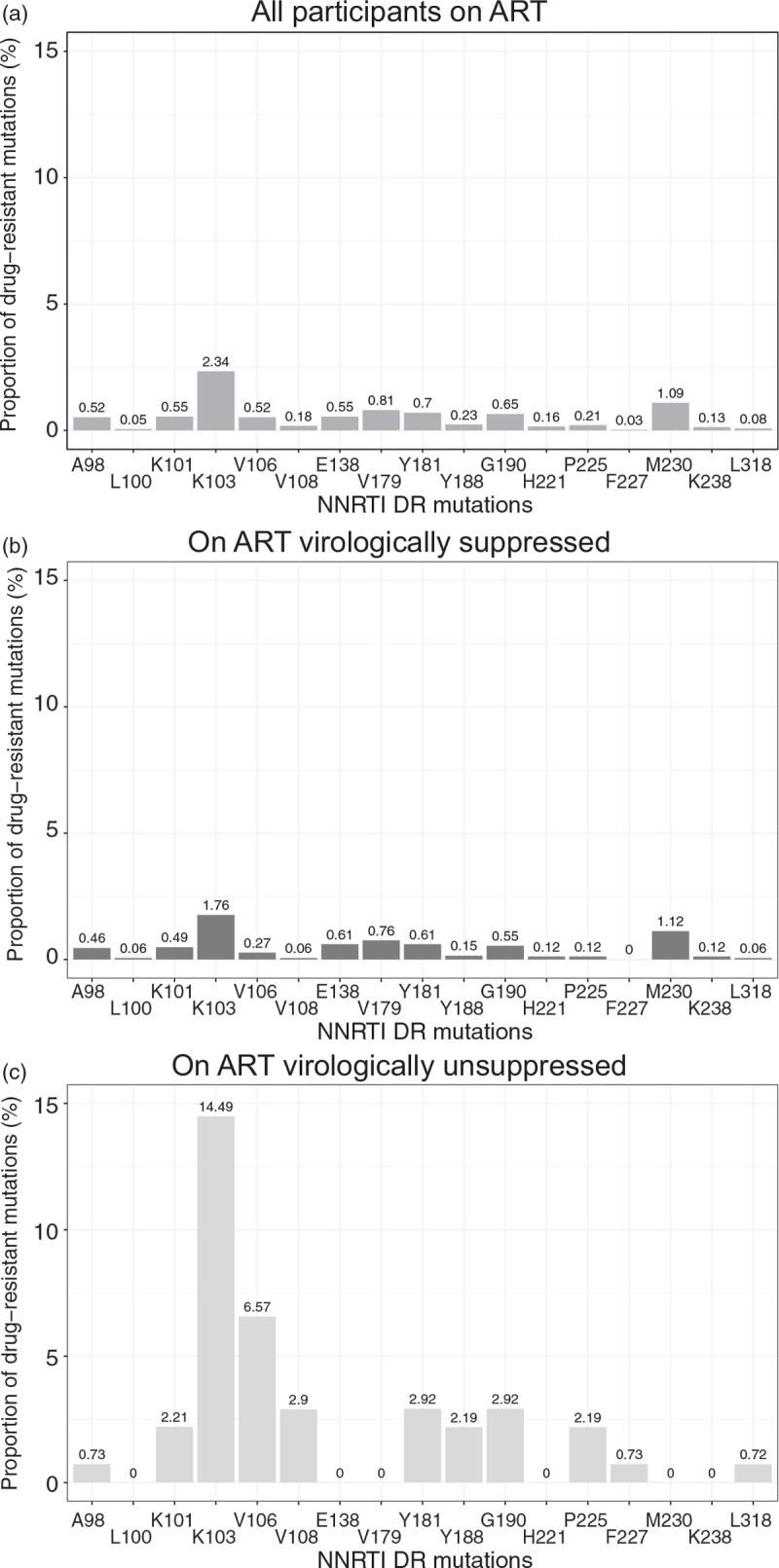
Proportion of nucleoside reverse transcriptase inhibitor drug resistant mutations among study participants on antiretroviral therapy.

Figures 2 and 3 illustrate the distribution of NRTI-associated and NNRTI-associated mutations within three subsets of BCPP participants receiving ART: All participants on ART (n = 3,435; Figs. 2a and 3a), participants on ART who are virologically suppressed (n = 3,297; Figs. 2b and 3b) and unsuppressed individuals receiving ART (*n* = 138; Figs. 2c and 3c).

The following amino acids substitutions were analyzed, as NRTI DRMs: E40F; M41L; E44D/A; A62V; K65R/E/N; D67N/G/E; T69D; K70R/E/G/Q/N/T; L74V/I; V75I; F77L; Y115F; F116Y; V118I; Q151M; M184I/V; L210W; T215Y/F/S/C/D/E/I/V/A/L/N; K219Q/E. (a) All participants on ART. (b) Participants on ART who are virologically suppressed (HIV-1 RNA ≤400 copies/ml). (c) Participants on ART who are virologically unsuppressed (HIV-1 RNA >400 copies/ml). ART, antiretroviral therapy; NRTI, nucleoside reverse transcriptase inhibitors; SDRM, surveillance drug resistant mutations.

The following amino acids substitutions were analyzed, as NNRTI DRMs: V90I; A98G; L100I; K101P/E/H; K103N/S; V106M/A/I; E138G/K/Q/R; V179D/F/T/L; Y181C/I/V; Y188L/H/C; G190A/S/E/Q; H221Y; P225H; F227C; M230L/I; K238T; L318F. (a) All participants on ART. (b) Participants on ART who are virologically suppressed (HIV-1 RNA ≤400 copies/ml). (c) Participants on ART who are virologically unsuppressed (HIV-1 RNA >400 copies/ml).

Distribution of NRTI-associated mutations among all individuals on ART (Fig. 2a) was similar to patterns seen among ART-naive individuals, although at a higher level. Thus, M184 mutations were observed at 2.2%, whereas M41, K65, D67, K70, T215 and K219 were seen between 0.16 and 0.49%. Among participants on ART and virologically suppressed, M184 (1.8%), was the most prevalent acquired drug resistance (ADR) mutation, whereas other mutations were less than 0.5% (Fig. 2b).

In contrast, individuals receiving ART with detectable levels of HIV-1 RNA (>400 copies/ml) demonstrated substantially higher levels of some, but not all NRTI-associated mutations (Fig. 2c). Prevalence of M184 mutations was 13.1%, whereas frequency of A62, K65, D67, K70, V75, and K219 mutations was in a range between 1.5 and 2.9%. Interestingly, no M41 or L210 mutations were observed within this subset of BCPP participants. Among 138 virologically unsuppressed individuals on ART, NRTI-associated mutations were found in 22 (15.9%) participants, who acquired one (11 participants), two (7 participants), three (3 participants) and four (1 participant; D67N, K70R, M184V and K219E) NRTI mutations.

The distribution of NNRTI-associated mutations within subsets of ART-experienced and virologically suppressed individuals on ART (Fig. 3b) resembled patterns observed for NRTI-associated mutations. DRMs at amino acid positions K103 and M230 were at the level of 1.8 and 1.1%, respectively, whereas other NNRTI-associated mutations among virologically suppressed BCPP participants on ART were below 1%.

Figure 3c depicts elevated prevalence of NNRTI-associated mutations among a subset of virologically unsuppressed individuals on ART. The most common NNRTI-associated mutation was at position K103 (14.5%), followed by mutation at amino acid position V106 (6.6%). With the exception of positions L100, E138, V179, H221, M230 and K238 (no mutations observed), other NNRTI-associated mutations within the subset of unsuppressed individuals on ART fluctuated between 0.72 and 2.9%. The prevalence ADR and/or SDRMs to NRTIs and NNRTIs did not differ by age, sex, time period or geographical region (Tables 1 and 2).

## Discussion

This is the first large, countrywide surveillance of HIVDR mutations to NRTI and NNRTI classes of antiretrovirals in Botswana during the era of expanded ART therapy. The key strength of this study is sampling that was well designed and performed by targeting predefined 20% of households in 30 rural and peri-urban communities across Botswana [13]. The second distinctive strength is the size of the study sample. The study data are based on analysis of HIV-1C polymerase gene sequences from 4973 participants including 1069 ART-naive individuals. The key finding is overall low prevalence of NRTI-associated and NNRTI-associated PDR in Botswana, 1.5 and 2.9%, respectively.

The study findings of low prevalence of pretreatment and acquired NRTI and NNRTI mutations are reassuring in light of maturing ART program and high ART coverage in Botswana. However, a recent report on sharp increase in transmitted drug resistance among recently infected pregnant women in one of the first ART sites in Botswana [12] suggests that targeted surveillance may be critical in certain regions of the country and among pregnant women.

Overall prevalence of PDR to NRTI or NNRTI falls within the range of the WHO's 5% threshold for a ‘low’ level of transmitted drug resistance [22]. The PDR was mostly driven by NNRTI-associated mutations found at 2.9%. The NRTI PDR accounted for 1.5%.

The NRTI-associated and NNRTI-associated DRMs were more prevalent in individuals currently on ART who were not virologically suppressed. Prevalence of NRTI-associated or NNRTI-associated DRMs among these individuals was 15.9 and 32.6%, respectively. These rates are lower than in Zambia (47.3%) or Cameroon (59.7%) according to the recent WHO report [23], and lower than the rates observed in rural and urban South Africa [24,25]. At the same time, a direct comparison between our data and other studies could be problematic because of difference in sampling (designed vs. convenience sampling) and sample size (large in BCPP vs. relatively small in other studies).

Results of our study highlight the importance of routine viral load testing and monitoring of HIVDR mutations associated with drug resistance at a population level during broad scale-up of ART including the treat all, as a national policy. Routine viral load monitoring and management of ART failure are in place in Botswana [26]. We also demonstrated that point-of-care viral load testing is feasible [27] and may provide rapid assessment of virologic failure.

The vast majority of BCPP participants in this study were on ART and had undetectable levels of HIV-1 RNA [13] precluding use of viral RNA as a reliable source for amplification. Therefore, in addition to viral RNA, we used proviral DNA as a template for amplification and sequencing. Previous studies including ours [28–31] demonstrated similarities between profiles of HIV DRMs derived from different host compartments. It would be interesting to compare drug resistance profiles in viral RNA and proviral DNA in a subset of participants on ART with detectable HIV-1 RNA in future studies. Clinical implications of HIV DRMs at low-level viremia or undetected viral load require further investigation especially in the era of highly potent ART regimens and high ART coverage. Emergence of DRMs in participants with undetectable viral load could predict virologic failure [30,32–35]. Monitoring of viral mutations combined with clinical and adherence data could reduce the likelihood of the appearance and spread of HIVDR [35,36].

The study has limitations. In this study, we focused on NRTI-associated and NNRTI-associated mutations, as the most commonly used antiretroviral classes in Botswana up to 2016. However, without the data on prevalence of DRMs to other antiretroviral classes, the picture of HIVDR landscape remains incomplete and warrants further studies. The individual ART regimens were not systematically collected in the BCPP study. As a population-based, BCPP was not designed to address the duration of ART or adherence to ART on individual level, as data collection and HIV testing was performed in households (not clinics). Therefore, the duration of ART and adherence data were not available. We could not rule out the possibility of undisclosed ART use among participants who self-reported not being on ART, as we showed recently [37]. This study was focused on analysis of single HIV sequence per participant represented by a consensus majority sequence from the NGS data. We detected virological failure without DRMs among HIV-infected individuals currently on ART and not suppressed. This may be primarily because of nonadherence or recent ART initiation or presence of minor DRMs. Minor DRMs were not analyzed in this study. Thus, focusing on dominant drug-resistant variants likely narrowed the true spectrum of circulating drug-resistant variants in Botswana.

In conclusion, distribution and prevalence of dominant HIV mutations associated with drug resistance to NRTI and NNRTI classes of antiretroviral drugs was addressed in a large, countrywide survey using a designed sampling across 30 rural and peri-urban communities in Botswana. Overall, low prevalence of dominant NRTI-associated and NNRTI-associated DRMs was found among ART-naive and ART-experienced participants. However, among a subset of virologically unsuppressed individuals receiving ART, prevalence of NRTI-associated and NNRTI-associated DRMs was above 15%. This study highlights the importance of monitoring and surveillance of HIVDR in response to scale-up of ART to inform public health policy and provide guidelines for optimal ART regimens.

## Acknowledgements

We thank greatly the study participants. We thank the BCPP staff and all field team members for their contribution to this study and making this study a success. We thank the Ministry of Health and Wellness and CDC Botswana for their excellent support and contributions to the study.

S.M., S.G. and V.N. conceived the study and prepared the first draft. E.v.W., E.R., M.P.H., K.E.W., M.E. and S.L. reviewed the manuscript and provided comments. S.M., V.N., S.G. and M.E. finalized the manuscript based on feedback from other authors. S.M., T.M., T.G., E.K., J.M., E.v.W. and K.E.W. collected or prepared the data. S.M., S.G., V.N., T.M., M.Z.S., D.C., T.D., O.B. and P.M. conducted and supervised the laboratory experiments. S.M., S.G. and V.N. analysed and interpreted the data. S.M., S.G., E.v.W., T.G., M.P.H., J.M.M., M.M., T.M., M.E. and S.L. helped provide overall guidance to the conduct of the study. M.P.H., J.M.M., M.M., V.N., S.M., S.G., M.E. and S.L. were involved in the origination and development of the concept of the study.

Funding: This study was supported by the United States President's Emergency Plan for AIDS Relief (PEPFAR) through the Centers for Disease Control and Prevention (CDC) under the terms of cooperative agreement U01 GH000447. The content is solely the responsibility of the authors and does not necessarily represent the official views of the funding agencies. S.M. was supported by the Fogarty International Center and National Institute of Mental Health, of the National Institutes of Health under Award Number D43 TW010543. S.M. and S.G. were partially supported by Sub-Saharan African Network for TB/HIV Research Excellence (SANTHE), a DELTAS Africa Initiative [grant # DEL-15-006]. The DELTAS Africa Initiative is an independent funding scheme of the African Academy of Sciences (AAS)'s Alliance for Accelerating Excellence in Science in Africa (AESA) and supported by the New Partnership for Africa's Development Planning and Coordinating Agency (NEPAD Agency) with funding from the Wellcome Trust [grant #107752/Z/15/Z] and the United Kingdom (UK) government. The views expressed in this publication are those of the authors and not necessarily those of PEPFAR, CDC, AAS, NEPAD Agency, Wellcome Trust, or the UK government. The funders had no role in the study design, data collection, and decision to publish, or in the preparation of the manuscript.

### Conflicts of interest

There are no conflicts of interest.

## Supplementary Material

Supplemental Digital Content
