# Supplementary material for: Low rates of nucleoside reverse transcriptase inhibitor and nonnucleoside reverse transcriptase inhibitor drug resistance in Botswana
Source: AIDS. 2019 Apr 4;33(6):1073–82. doi: 10.1097/QAD.0000000000002166 (PMC6467559; doi:10.1097/QAD.0000000000002166)
Supplement: Supplemental Digital Content [file aids-33-1073-s001.docx]

Supplementary Table S1. Relevance to hypermutation of selected amino acid mutations associated with HIV-1 drug resistance.

| Wild type Amino Acid aa | Wild aa type encoding | Mutated Amino Acid | Mutated aa encoding | Relevance to hypermutation | Comment |
| --- | --- | --- | --- | --- | --- |
| A | gca/gcc/gcg/gct | G | gga/ggc/ggg/ggt | not relevant |  |
|  |  | I | ata/atc/att | not relevant | requires 2 step mutations: not considered for Hypermutation adjustment |
|  |  | L | ctt/ctc/cta/ctg/tta/ttg | not relevant |  |
|  |  | **T** | aca/acc/acg/act | **relevant** |  |
|  |  | V | gta/gtc/gtg/gtt | not relevant | gcg to gta requires 2 step mutations: not considered for Hypermutation adjustment |
| D | gac/gat | E | gaa/gag | not relevant |  |
|  |  | G | gga/ggc/ggg/ggt | not relevant |  |
|  |  | **N** | aac/aat | **relevant** |  |
| E | gaa/gag | A | gca/gcc/gcg/gct | not relevant |  |
|  |  | G | gga/ggc/ggg/ggt | not relevant |  |
|  |  | **K** | aaa/aag | **relevant** |  |
|  |  | Q | caa/cag | not relevant |  |
|  |  | R | cga/cgc/cgg/cgt/aga/agg | not relevant |  |
| F | ttc/ttt | C | tgc/tgt | not relevant |  |
|  |  | L | ctt/ctc/cta/ctg/tta/ttg | not relevant |  |
|  |  | Y | tac/tat | not relevant |  |
| G | gga/ggc/ggg/ggt | A | gca/gcc/gcg/gct | not relevant |  |
|  |  | C | tgc/tgt | not relevant |  |
|  |  | **E** | gaa/gag | **relevant** |  |
|  |  | R | cga/cgc/cgg/cgt | not relevant |  |
|  |  | **R** | aga/agg | **relevant** |  |
|  |  | S | tca/tcc/tcg/tct | not relevant |  |
|  |  | **S** | agc/agt | **relevant** |  |
|  |  | T | aca/acc/acg/act | not relevant | requires 2 step mutations: not considered for Hypermutation adjustment |
|  |  | V | gta/gtc/gtg/gtt | not relevant |  |
| H | cac/cat | Y | tac/tat | not relevant |  |
| I | ata/atc/att | A | gca/gcc/gcg/gct | not relevant |  |
|  |  | L | ctt/ctc/cta/ctg/tta/ttg | not relevant |  |
|  |  | M | atg | not relevant |  |
|  |  | S | tca/tcc/tcg/tct/agc/agt | not relevant |  |
|  |  | T | aca/acc/acg/act | not relevant |  |
|  |  | V | gta/gtc/gtg/gtt | not relevant |  |
| K | aaa/aag | E | gaa/gag | not relevant |  |
|  |  | H | cac/cat | not relevant |  |
|  |  | I | ata/atc/att | not relevant |  |
|  |  | M | atg | not relevant |  |
|  |  | N | aac/aat | not relevant |  |
|  |  | P | ccc/cct | not relevant |  |
|  |  | Q | caa/cag | not relevant |  |
|  |  | R | cga/cgc/cgg/cgt/aga/agg | not relevant |  |
|  |  | S | tca/tcc/tcg/tct/agc/agt | not relevant |  |
|  |  | T | aca/acc/acg/act | not relevant |  |
|  |  | V | gta/gtc/gtg/gtt | not relevant |  |
| L | ctt/ctc/cta/ctg/tta/ttg | C | tgc/tgt | not relevant |  |
|  |  | F | ttc/ttt | not relevant |  |
|  |  | I | ata/atc/att | not relevant |  |
|  |  | M | atg | not relevant |  |
|  |  | P | ccc/cct | not relevant |  |
|  |  | R | cga/cgc/cgg/cgt/aga/agg | not relevant |  |
|  |  | V | gta/gtc/gtg/gtt | not relevant |  |
|  |  | W | tgg | not relevant |  |
| M | atg | **I** | ata | **relevant** |  |
|  |  | I | atc/att | not relevant |  |
|  |  | L | ctt/ctc/cta/ctg/tta/ttg | not relevant |  |
|  |  | V | gta/gtc/gtg/gtt | not relevant |  |
| N | aac/aat | D | gac/gat | not relevant |  |
|  |  | H | cac/cat | not relevant |  |
|  |  | S | tca/tcc/tcg/tct/agc/agt | not relevant |  |
| P | ccc/cct | H | cac/cat | not relevant |  |
| Q | caa/cag | H | cac/cat | not relevant |  |
|  |  | K | aaa/aag | not relevant |  |
|  |  | M | atg | not relevant |  |
|  |  | R | cga/cgc/cgg/cgt/aga/agg | not relevant |  |
| R | cga/cgc/cgg/cgt/aga/agg | **K** | aaa/aag | **relevant** |  |
| S | tca/tcc/tcg/tct/agc/agt | G | gga/ggc/ggg/ggt | not relevant |  |
| T | aca/acc/acg/act | A | gca/gcc/gcg/gct | not relevant |  |
|  |  | F | ttc/ttt | not relevant |  |
|  |  | I | ata/atc/att | not relevant |  |
|  |  | K | aaa/aag | not relevant |  |
|  |  | P | ccc/cct | not relevant |  |
|  |  | Y | tac/tat | not relevant |  |
| V | gta/gtc/gtg/gtt | A | gca/gcc/gcg/gct | not relevant |  |
|  |  | C | tgc/tgt | not relevant |  |
|  |  | D | gac/gat | not relevant |  |
|  |  | F | ttc/ttt | not relevant |  |
|  |  | **I** | ata/atc/att | **relevant** |  |
|  |  | L | ctt/ctc/cta/ctg/tta/ttg | not relevant |  |
|  |  | **M** | atg | **relevant** |  |
|  |  | S | tca/tcc/tcg/tct/agc/agt | not relevant | requires 2 step mutations: not considered for HM adjustment |
|  |  | T | aca/acc/acg/act | not relevant | requires 2 step mutations: not considered for HM adjustment |
| Y | tac/tat | C | tgc/tgt | not relevant |  |
|  |  | F | ttc/ttt | not relevant |  |
|  |  | H | cac/cat | not relevant |  |
|  |  | I | ata/atc/att | not relevant |  |
|  |  | L | ctt/ctc/cta/ctg/tta/ttg | not relevant |  |
|  |  | R | cga/cgc/cgg/cgt/aga/agg | not relevant |  |
|  |  | V | gta/gtc/gtg/gtt | not relevant |  |

Amino acid codes and Abbreviations: A - Alanine (Ala); R - Arginine (Arg); N - Asparagine (Asn); D - Aspartic acid (Asp); B - Asn or Asp (Asx); C - Cysteine (Cys); Q - Glutamine (Gln); E - Glutamic acid (Glu); Z - Gln or Glu (Glx); G - Glycine (Gly); H - Histidine (His); I - Isoleucine (Ile); L - Leucine (Leu); K - Lysine (Lys); M - Methionine (Met); F - Phenylalanine (Phe); P - Proline (Pro); S - Serine (Ser); T - Threonine (Thr); W - Tryptophan (Trp); Y - Tyrosine (Tyr); V - Valine (Val)
